# Development of Quality of Life in Adolescents and Young Adults With Cancer Using a Patient Support Smartphone App: Prepost Interventional Study

**DOI:** 10.2196/49735

**Published:** 2023-12-04

**Authors:** Line Bentsen, Signe Hanghøj, Maiken Hjerming, Mette Buur Bergmann, Marianne Thycosen, Anette Borup, Camilla Larsen, Helle Pappot

**Affiliations:** 1 Department of Oncology Copenhagen University Hospital, Rigshospitalet Copenhagen Denmark; 2 Department of Haematology Copenhagen University Hospital, Rigshospitalet Copenhagen Denmark; 3 Department of Haematology Zealand University Hospital Roskilde Denmark; 4 Department of Oncology Odense University Hospital Odense Denmark; 5 Department of Blood Diseases Aarhus University Hospital Aarhus Denmark; 6 Department of Haematology Aalborg University Hospital Aalborg Denmark

**Keywords:** adolescent, young adult, cancer, quality of life, eHealth, smartphone application, application, development, interventional study, youth, grief, symptom tracker, social community, Denmark, physical functioning, treatment, mobile phone

## Abstract

**Background:**

Adolescents and young adults often experience existential concerns in addition to side effects during a cancer trajectory, which they often carry alone. Thus, cohesion with other adolescents and young adults with cancer is essential but difficult due to the relatively small, widely dispersed nationwide population. In cocreation, a smartphone app has been developed and includes an information bank, a symptom tracker, and a social community platform, aiming to improve the quality of life (QoL) in this patient group.

**Objective:**

This nationwide, multicenter study aimed to investigate the QoL in adolescents and young adults undergoing a cancer trajectory as they used the app for 6 weeks.

**Methods:**

Via youth support initiatives, participants were recruited from hospitals in all regions of Denmark. Inclusion criteria were patients with cancer aged 15-29 years who either initiated any cancer treatment or started follow-up after cancer treatment within 30 days. Participants used the adolescents and young adults cancer app for 6 weeks. Before and after the 6 weeks of app use, they completed the European Organization for Research and Treatment of Cancer Quality of Life Questionnaire Core 30 (EORTC QLQ-C30). The participants were divided into a treatment and a follow-up group for analysis. A high score for a functional scale or the global health or overall QoL represents a high or healthy level of functioning or high QoL, respectively; however, a high score for a symptom scale or item represents a high level of symptomatology.

**Results:**

Overall, 81 participants were recruited. However, 4 participants did not answer the questionnaire and 6 participants did not use the app. In the treatment group (n=36), significant improvement was found in 2 domains: “Role functioning” (baseline median 33.33, IQR 16.67-83.33 vs 6 weeks median 66.67, IQR 33.33-83.33; *P*=.04) and “Pain” (baseline median 33.33, IQR 16.67-50.00 vs 6 weeks median 16.67, IQR 0.00-33.33; *P*=.04). The “Global health/Overall QoL” scale remained stable (baseline median 58.33, IQR 45.83-77.08 vs 6 weeks median 62.50, IQR 41.67-75.00; *P*=.25). In the follow-up group (n=35), significant improvement was found in 3 domains: “Physical functioning” (baseline median 79.23, IQR 73.33-93.33 vs 6 weeks median 82.86, IQR 73.33-100.00; *P*=.03), “Cognitive functioning” (baseline median 62.38, IQR 50.00-83.33 vs 6 weeks median 69.52, IQR 50.00-100.00; *P*=.02), and “Social functioning” (baseline median 76.19, IQR 50.00-100.00 vs 6 weeks median 85.71, IQR 83.33-100.00; *P*=.05), as well as in the “Global health/Overall QoL” scale (baseline median 57.14, IQR 83.33-100.00 vs 6 weeks median 75.0, IQR 62.91-85.73; *P*<.001).

**Conclusions:**

In this study, we found an improvement in specific QoL scales for both participants in treatment and follow-up when using the app for 6 weeks. The global health or overall QoL score improved significantly in the follow-up group. In the treatment group, it remained stable.

**International Registered Report Identifier (IRRID):**

RR2-10.2196/10098

## Introduction

Each year, 600 adolescents and young adults aged 15-29 years are diagnosed with cancer in Denmark. Further, an increase in young cancer survivors exists due to cancer detection improvement and advanced cancer therapy [1,2]. Adolescents and young adults with cancer and young cancer survivors represent a unique patient group undergoing identity development during the cancer trajectory [3]. They are, therefore, vulnerable to identity and existential concerns. The concerns can leave them anxious and loaded with grief, which they often carry alone. Cohesion with other adolescents and young adults is essential, for example, through modern technological solutions such as online networks [4-7]. It is known that adolescents and young adults in cancer treatment often experience a burdensome trajectory, accumulating several symptoms during treatment, and it is not uncommon that quality of life (QoL) decreases [8,9].

User involvement and patient-reported outcome (PRO) are new perspectives implemented in scientific projects in recent years, focusing on aspects from patients’ points of view [10-13]. The Danish smartphone app, “Kræftværket,” aimed at adolescents and young adults with cancer, was created based on user involvement and by request of the users [5,14]. The app was developed to improve the QoL across diagnosis, gender, and age subgroups among adolescents and young adults [14]. It was designed by and for adolescents and young adults with cancer in 2018 and was implemented nationally as part of a research project in 2019 with great success [15]. Currently, 650 adolescents and young adults are active on the adolescents and young adults cancer app platform. The users are a mix of adolescents and young adults in active cancer treatment and adolescents and young adults in follow-up. The platform includes a tracking module focusing on symptoms and activities, an information bank including both text and video material, and a social community platform that facilitates networking and sharing experiences. Tracking symptoms using PROs during cancer trajectories has been shown to improve QoL [16].

The feasibility of using the adolescents and young adults cancer app has previously been demonstrated [5]. Further, a pilot study assessing the QoL at baseline and after 6 weeks of use of the same app was conducted in a very small population unaligned to the start of the treatment [14]. Preliminary results from the single-center pilot study suggested a possible positive effect when using the adolescents and young adults cancer app during cancer treatment and follow-up [17]. Still, a confirmatory study in a larger, national population is needed.

The aim of this national, multicenter study was to investigate QoL in a more extensive and aligned population with adolescents and young adults in a cancer trajectory as they used the adolescents and young adults cancer app for 6 weeks.

## Methods

### Participants

Eligible patients were adolescents and young adults aged 15-29 years who, within 30 days, either were (1) diagnosed with cancer and starting any cancer treatment or (2) starting follow-up after cancer treatment.

Inclusion criteria were adolescents and young adults aged 15-29 years with access to smartphones and the internet, including cellular data or Wi-Fi. Exclusion criteria were participating in the cocreation process and inability to read and write in Danish.

### Study Setting and Recruitment

The study is anchored at the Departments of Oncology, Haematology, and Paediatrics and Adolescent Medicine at the Copenhagen University Hospital, Rigshospitalet, in the youth support center, Kræftværket. The study is national, including all regions in Denmark. Recruitment took place at the Department of Oncology at Odense University Hospital; the Department of Blood Diseases at Aarhus University Hospital; the Departments of Haematology at Zealand University Hospital, Roskilde, and Aalborg University Hospital; and the Department of Oncology and Haematology at Copenhagen University Hospital, Rigshospitalet.

Before recruitment, the distribution of adolescents and young adults in the respective regions was calculated to secure equality. The estimated recruitment was calculated to: 35% (35/100) of patients from the Capital Region of Denmark (Copenhagen University Hospital, Rigshospitalet), 21% (21/100) of patients from the Region of Southern Denmark (Odense University Hospital), 21% (21/100) of patients in the Central Denmark Region (Aarhus University Hospital), 14% (14/100) of patients from the Region Zealand (Zealand University Hospital, Roskilde), and 9% (9/100) of patients from the North Denmark Region (Aalborg University Hospital).

Participants were recruited from September 14, 2020, through May 2022, and a convenience sampling method was adopted. Potentially 600 individuals were recruitable each year from these departments; however, no screening log was used in this study.

Nurse specialists and adolescents and young adults youth coordinators sought to invite all adolescents and young adults undergoing a cancer trajectory to one of the abovementioned departments. Recruitment strategies also included advertisement in closed groups on social media such as Facebook and at social events at the youth facility centers.

Verbal and written study information was provided to eligible patients within the hospitals by the adolescents and young adults youth coordinators. The youth coordinators help adolescents and young adults with cancer navigate the health care system through their cancer trajectory and manage social events and activities in the existing social support groups. The youth coordinators also obtained informed consent. Finally, they helped with app information and installation guides and were available if the participants had questions about the study or needed support for the app.

### Intervention

The intervention in this study was a health app specially developed to improve the QoL in adolescents and young adults with cancer. The Kræftværket app has three primary features: (1) a symptom and activity diary, (2) a supportive communication network between app users, and (3) a “one-stop shop” information bank with practical information as well as links to patient organizations and other resources.

The intervention requires that adolescents and young adults use the app over the course of 6 weeks and complete a baseline and follow-up European Organization for Research and Treatment of Cancer Quality of Life Questionnaire Core 30 (EORTC QLQ-C30) health-related QoL inventory. Further information on the app’s features and modules has previously been published [14]. The 6-week app use period was decided based on a comparable study including Danish adolescents and young adults with diabetes showing that the number of young people using the eHealth tool decreased after 6 weeks [18]. Both Android and iPhone operating systems were compatible with the intervention app.

### Questionnaires

The participants were asked to complete 2 questionnaires. The first was the validated QoL EORTC QLQ-C30 (version 3.0) questionnaire [19-21]. This was used to assess the QoL at baseline and after 6 weeks of use of the adolescents and young adults cancer app. The EORTC QLQ-C30 questionnaire consists of functional scales, a global health or overall QoL scale, and symptom scales. A high score for a functional scale or the global health or overall QoL represents a high or healthy level of functioning or high QoL, respectively. On the contrary, a high score for a symptom scale or item represents a high level of symptomatology. For statistical analysis, scoring the EORTC QLQ-C30 scales is necessary, and the *EORTC QLQ-C30 Scoring Manual* was used for this purpose [22].

The second questionnaire focused on the experience of the social community platform available in the app. The results on the experience of the social community platform are reported elsewhere [23].

### Statistical Analysis

Our study was initially inspired by studies performed in populations with breast cancer for testing apps as interventions for improving QoL and well-being. These studies comprised 30 to 78 participants [24] and investigated an app in adolescents and young adults with diabetes [25,26].

Since the minimum important difference being clinically meaningful for EORTC QLQ-C30 in adolescents and young adults with cancer undergoing therapy and in follow-up was not known at the time of designing the study, the intended sample size of 100 was solely based on experience from other similar studies.

The raw data from the EORTC QLQ-C30 questionnaires were entered into an Excel file (Microsoft Office version 2018) and imported to the statistical software program R (version 1.4.1717; Lucent Technologies). Using the *PRO Score* package, the raw data was transformed into the global health or overall QoL scale, the functional scales, and the symptom scales [22].

We examined for normal distribution within each scale with histograms and quantile-quantile plots. Since no scales were normally distributed, we used the Wilcoxon signed rank exact test to determine potential differences from baseline and after 6 weeks. Data from the participants were divided into 2 groups: participants in cancer treatment and participants in follow-up. All participants were included in the descriptive statistical analysis and baseline analysis. Participants in both groups who did not fill out the questionnaire after 6 weeks or did not use the app during the study period were not included in the statistical analysis of the EORTC QLQ-C30 scores.

### Ethical Considerations

As from May 26, 2021, ethical approval in intervention studies with smartphone apps using QoL-questionnaires as outcome were mandatory according to the Danish ethical committee [27]. However, the initiation of this study was prior to this date; thus, this study was exempt from ethical approval according to Danish law. Data approval from the Danish Data Protection Agency (P-2020-317) was achieved before recruitment. Written informed consent was obtained from all study participants. In cases where the participant was younger than 18 years, the caretaker’s consent was obtained.

In this study, the data are anonymous. Participants did not receive compensation.

## Results

### Recruitment

In total, 85 participants were recruited for the study. Further, 2 participants were excluded, and another 2 participants withdrew their informed consent. We did not receive the EORTC QLQ-C30 questionnaire after 6 weeks from 4 participants: 2 from the treatment group and 2 from the follow-up group. The reasons for this were that 1 died, 1 had problems with the app, 1 was too ill, and 1 was unknown. Further, 6 participants did not use the app: 5 from the treatment group and 1 from the follow-up group. However, 4 of the 6 participants still answered the EORTC QLQ-C30 questionnaire at baseline and after 6 weeks. The reasons why the participants did not use the app were that some of the participants were too ill (n=3), were in shock over their cancer diagnosis (n=2), or did not need the app (n=1).

**Figure 1:**
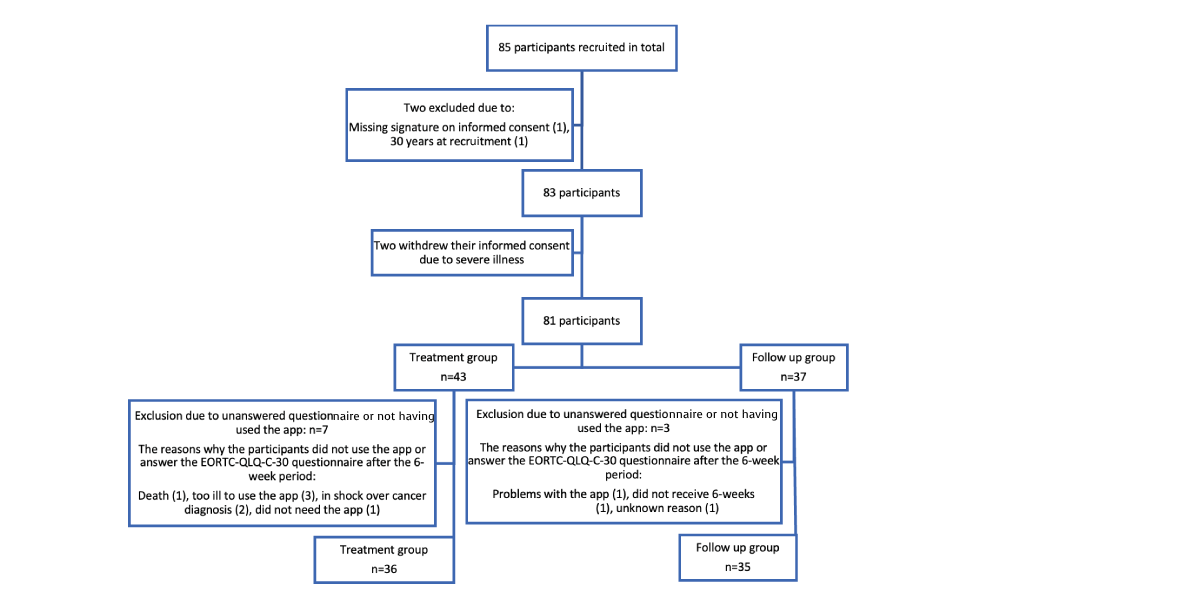
Flowchart showing recruitment of participants and reasons of exclusion. EORTC QLQ-C30: European Organization for Research and Treatment of Cancer Quality of Life Questionnaire Core 30.

Participants who did not fill out the questionnaire after 6 weeks and those who did not use the app were excluded from the final comparison analysis. After exclusion, the participants were equally distributed, with 36 participants in the treatment group and 35 in the follow-up group (Figure 1). Recruitment was achieved by the distribution estimation (Table 1).

**Table 1:** Regional distribution of participants.

Recruitment region	Estimated participants (n=100), n (%)	Recruited participants (n=81), n (%)
The Capital Region of Denmark (Copenhagen University Hospital, Rigshospitalet)	35 (35)	34 (42)
Region Zealand (Zealand University Hospital, Roskilde)	14 (14)	10 (12)
Region of Southern Denmark (Odense University Hospital)	21 (21)	15 (18)
Central Denmark Region (Aarhus University Hospital)	21 (21)	13 (16)
North Denmark Region (Aalborg University Hospital)	9 (9)	9 (11)
Total	100 (100)	81 (100)

### Characteristics in the Study Population

Demographic data showed equal distribution of gender, with a slightly overweight of women compared to men in both the treatment and the follow-up group. Additionally, age and age range were similar in both groups. Clinical data showed a representation of same cancer types in both groups except that gastrointestinal cancer (n=2), thyroid cancer (n=1), and uterus cancer (n=1) appeared in the treatment group only. In the follow-up group, central nervous system cancer (n=2), neuroendocrine tumor (n=1), and malign melanoma (n=1) appeared (Table 2).

**Table 2:** Demographic and clinical data of the participants.

Characteristics		Treatment group (n=36)	Follow-up group (n=35)
**Gender, n (%)**			
	Men	16 (44)	16 (46)
	Women	20 (56)	19 (54)
**Age (y), median (range)**		24 (18-29)	23 (18-29)
**Cancer type, n (%)**			
	Lymphoma	15 (42)	11 (31)
	Breast	1 (3)	2 (6)
	Leukemia	5 (14)	5 (14)
	Testicular	7 (19)	7 (20)
	GI^a^	2 (6)	0 (0)
	Thyroid	1 (3)	0 (0)
	CNS^b^	0 (0)	2 (6)
	NET^c^	0 (0)	1 (3)
	Sarcoma	1 (3)	3 (9)
	Ovarian	2 (6)	2 (6)
	Cervix	1 (3)	1 (3)
	Malignant melanoma	0 (0)	1 (3)
	Uterus	1 (3)	0 (0)

^a^GI: gastrointestinal.

^b^CNS: central nervous system.

^c^NET: neuroendocrine tumor.

### EORTC QLQ-C30 Scores

In the treatment group, significant change was found in 2 domains from baseline to after 6 weeks. Both in the domain “Role functioning” (baseline median 33.33, IQR 16.67-83.33 vs 6 weeks median 66.67, IQR 33.33-83.33; *P*=.04) and the domain “Pain” (baseline median 33.33, IQR 16.67-50.00 vs 6 weeks median 16.67, IQR 0.00-33.33; *P*=.04), the score was improved after 6 weeks (Table 3).

**Table 3:** EORTC QLQ-C30^a^ scores in the treatment group.

Treatment group (n=36)	Baseline, median (IQR)	6 weeks, median (IQR)	Wilcoxon pseudo median (95% CI)	Wilcoxon *P* value (<.05)
Global health or overall QoL^b^	58.33 (45.83-77.08)	62.50 (41.67-75.00)	–4.49 (–13.08 to 4.00)	.25
Physical functioning	80.00 (60.00-93.33)	80.00 (73.33-88.33)	0.71 (–6.69 to 6.83)	.57
Role functioning	33.33 (16.67-83.33)	66.67 (33.33-83.33)	–15.34 (–25.67 to –0.63)	.04
Emotional functioning	70.83 (58.33-83.33)	66.67 (58.33-83.33)	0.61 (–5.12to 8.96)	.72
Cognitive functioning	83.33 (62.50-100.00)	83.33 (50.0-83.33)	2.56 (–1.88 to 10.18)	.29
Social functioning	66.67 (50.00-83.33)	66.67 (50.0-83.33)	0.63 (–8.02 to 9.12)	.70
Fatigue	55.56 (33.33-77.78)	55.56 (33.33-80.56)	0.33 (–10.80 to 11.23)	.81
Nausea or vomiting	16.67 (0.00-33.33)	0.00 (0.00-20.83)	7.43 (–0.28 to 15.18)	.09
Pain	33.33 (16.67-50.00)	16.67 (0.00-33.33)	16.66 (0.974 to 26.14)	.04
Dyspnea	16.67 (0.00-33.33)	0.00 (00.00-33.33)	1.54 (–2.44 to 15.10)	.32
Insomnia	33.33 (0.00-66.67)	33.33 (25-41.67)	–1.28 (–16.82 to 14.61)	.68
Appetite loss	33.33 (0.00-66.67)	00.00 (0.00-33.33)	13.32 (–3.30 to 31.75)	.32
Constipation	00.00 (0.00-33.33)	00.00 (0.00-33.33)	–1.29 (–17.40 to 14.54)	.68
Diarrhea	00.00 (0.00-33.33)	00.00 (0.0-33.33)	2.16 (–2.10 to 15.48)	.46
Financial difficulties	00.00 (0.00-33.33)	00.00 (0.00-33.33)	–0.35 (–4.032 to 2.79)	.77
QLQ-C30^c^ summary score	71.71 (54.62-80.32)	74.17 (62.30-84.83)	–3.75 (–10.54 to 3.16)	.29

^a^EORTC QLQ-C30: European Organization for Research and Treatment of Cancer Quality of Life Questionnaire Core 30.

^b^QoL: quality of life.

^c^QLQ-C30: Quality of Life Questionnaire Core 30.

In the follow-up group, significant change was found in 3 functional domains from baseline and after 6 weeks. In all 3 domains, “Physical functioning” (baseline median 79.23, IQR 73.33-93.33 vs 6 weeks median 82.85, IQR 73.33-100.00; *P*=.03), “Cognitive functioning” (baseline median 62.38, IQR 50.00-83.33 vs 6 weeks median 69.52, IQR 50.00-100.00; *P*=.02), and “Social functioning” (baseline median 76.19, IQR 66.67-100.00 vs 6 weeks median 85.71, IQR 83.33-100.00; *P*=.046), the score was improved after 6 weeks. Further, the “Global health/overall QoL” scale enhanced over time (baseline median 57.14, IQR 41.67-66.67 vs 6 weeks median 75.0, IQR 54.17-83.33; *P*<.001). A significant change was also seen in the “QLQ-C30 summary score” (baseline median 74.95, IQR 62.91-85.73 vs 6 weeks median 79.41, IQR 62.91-85.73; *P*=.03; Table 4).

**Figure 2:**
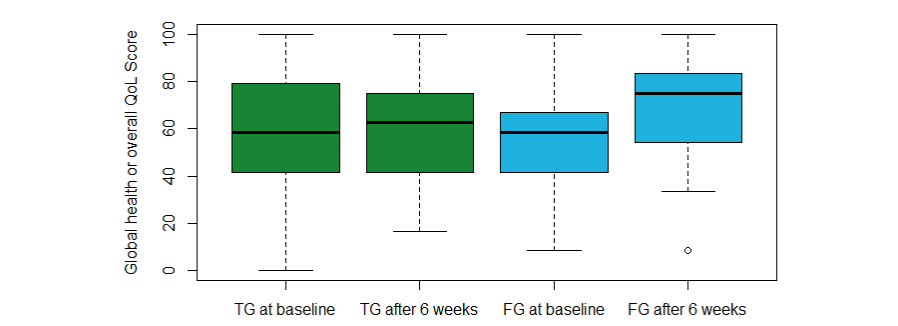
Boxplot showing the global health or overall QoL score at baseline and after 6 weeks in the TG and in the FG. FG: follow-up group; QoL: quality of life; TG: treatment group.

In the follow-up group, a significant change was found in the score “Global health/overall QoL.” In contrast, the insignificant score in the treatment group (Figure 2).

**Table 4:** EORTC QLQ-C30^a^ scores in the follow-up group.

Follow-up group (n=35)	Baseline, median (IQR)	6 weeks, median (IQR)	Wilcoxon pseudo median (95% CI)	Wilcoxon *P* value (<.05)
Global health or overall QoL^b^	57.14 (41.67-66.67)	75.00 (54.17-83.33)	–8.99 (–16.56 to –4.66)	<.001
Physical functioning	79.24 (73.33-93.33)	82.86 (73.33-100.00)	–3.56 (–6.81 to –0.094)	.03
Role functioning	66.67 (50.00-91.67)	73.81 (58.33-100.00)	–5.54 (–17.42 to 6.02)	.27
Emotional functioning	65.47 (50.00-83.33)	72.38 (62.50-87.50)	–4.84 (–12.14 to 0.13)	.07
Cognitive functioning	62.38 (50.00-83.33)	69.52 (50.00-100.00)	–8.48 (–16.20 to –0.70)	.02
Social functioning	76.19 (66.67-100.00)	85.71 (83.33-100.00)	–8.25 (–17.12 to –0.06)	.046
Fatigue	46.03 (22.22-66.67)	39.05 (22.22-50.00)	6.49 (–0.027 to 14.60)	.05
Nausea or vomiting	12.38 (0.00-16.67)	10.00 (0.00-16.67)	0.69 (–0.72 to 2.23)	.22
Pain	21.90 (0.00-41.67)	19.05 (0.00-33.33)	1.39 (–2.35 to 10.44)	.37
Dyspnea	14.29 (0.00-33.33)	12.38 (0.00-33.33)	0.57 (–2.75 to 11.53)	.69
Insomnia	39.05 (16.67-66.67)	35.24 (0.00-66.67)	1.86 (–1.16 to 11.40)	.23
Appetite loss	20.95 (0.00-33.33)	17.14 (0.00-33.33)	–0.48 (–4.71 to 4.01)	.76
Constipation	13.33 (0.00-16.67)	9.52 (0.00-16.67)	0.56 (–2.16 to 3.55)	.67
Diarrhea	7.62 (0.00-16.66)	9.52 (0.00-16.67)	–1.07 (–3.38 to 0.82)	.23
FI: financial difficulties	16.19 (0.00-33.33)	17.14 (0.00-33.33)	–0.75 (–3.46 to 4.28)	.61
QLQ-C30^c^ summary score	74.95 (62.91-85.73)	79.41 (71.65-90.38)	–3.47 (–7.08 to –0.37)	.03

^a^EORTC QLQ-C30: European Organization for Research and Treatment of Cancer Quality of Life Questionnaire Core 30.

^b^QoL: quality of life.

^c^QLQ-C30: Quality of Life Questionnaire Core 30.

## Discussion

### Principal Findings

In this study, we found an improvement in specific QoL scales for both adolescents and young adults in treatment and adolescents and young adults in follow-up when using the app for 6 weeks.

Significant improvement from baseline throughout the 6 weeks was found in the follow-up group related to the physical, cognitive, and social functions, as well as for role functioning and pain in the treatment group. In the follow-up group, the global health or overall QoL score improved significantly. In the treatment group, it remained stable.

These results support the findings from the more unstructured and smaller pilot study by Pappot et al [17], where QoL was first tested when using the same adolescents and young adults cancer app. As in this study, the pilot study found a significant increase in overall QoL after 6 weeks in the follow-up group. For the treatment group, the QoL remained stable throughout the 6 weeks. This initial finding could have been due to the single-center nature of the study. This study is strengthened by the fact that the app as an intervention was developed in a single institution and tested in a national, multicenter study. Participation in the cocreation process was an exclusion criterion for this study.

Adolescents and young adults in cancer treatment often experience a burdensome trajectory, accumulating several symptoms during treatment, and it is not uncommon that QoL decreases [8,9]. In this study, no decrease in QoL after 6 weeks is seen despite the expectation of increasing symptom burden during treatment. The stable level of the overall QoL among adolescents and young adults in treatment is therefore interpreted as a positive result.

In addition, patients with hematological diagnoses were overrepresented in the cancer treatment group compared to the follow-up group (20/36, 56% vs 16/35, 45%), which could influence the symptom frequency negatively due to severe symptoms during treatment among hematological patients [28]. Therefore, the significant improvement in the symptom domain “Pain” is interesting, since pain is often sustained during cancer treatment [8]. However, the improvement might be explained by adolescents and young adults cancer app use, as previously suggested, within patients with cancer and chronic pain [29-31].

Among adolescents and young adults in cancer treatment, results show significant improvement in the domain of “Role functioning.” This can be explained not by the effect of using the adolescents and young adults cancer app only but by the phenomenon of “Response shift,” where the meaning of an individual’s self-evaluation of their health status and QoL change over time [32]. Adolescents and young adults in cancer treatment are likely to transition from experiencing initial crisis and loss of control at diagnosis to accepting their diagnosis and situation. An increase in awareness and use of coping strategies supports this transition in this patient group [33].

Several others have demonstrated that QoL is reported to decrease compared to the background population, especially regarding the social, emotional, cognitive, role, and physical functions [34,35]. It is essential to notice that Husson et al [34] found no difference in QoL among short- and long-term lymphoma survivors. Furthermore, Geue et al [35] found that adolescents and young adults had reduced QoL in comparison with the general population even a long time after the treatment was completed, where female adolescents and young adults with cancer reported significantly lower QoL compared to male adolescents and young adults with cancer. This argues the need for great awareness of the risk of a long-term decrease in QoL among adolescents and young adults survivors after cancer and how to prevent this [7,36]. Significant increase in several functional domains were found for both the treatment and follow-up groups in our study, suggesting that adolescents and young adults cancer apps could benefit this patient group during cancer treatment and follow-up. The results in our study mirror the findings of others related to the association between smartphone app use and QoL in young patients with cancer. The pilot randomized clinical trial by El-Jawari et al [37] found that their psychological smartphone app for young, newly diagnosed patients with acute myeloid leukemia showed significant improvement in QoL (Functional Assessment of Cancer Therapy-Leukemia) and self-efficacy (Cancer Self-Efficacy Scale) and a decrease in symptom burden (Edmonton Symptom Assessment Scale) in patients using the smartphone app compared to the patients receiving standard care. Additionally, the meta-analysis by Siew et al [38] demonstrated that social media–based interventions significantly improved QoL and anxiety symptoms among patients with cancer.

A possible reason contributing to the significant increase in QoL in the follow-up group in our study could be that the smartphone app enabled a community with equals outside the hospital and beyond the cancer trajectory. The qualitative interviews conducted in our study population showed that some adolescents and young adults assessed the social forum as more valuable at diagnosis, while others experienced meaningful peer support after cancer [23]. Lea et al [39] also found in their qualitative study that adolescents and young adults with cancer were underprepared for and challenged by the unexpected, emotional, and physical consequences of ending cancer treatment. By providing an online community, the smartphone app could potentially bridge the transition from ending cancer treatment into the follow-up trajectory for adolescents and young adults with cancer.

### Strengths and Limitations

A limitation of this study is that we do not have data linking time consumption at each of the 4 modules in the adolescents and young adults cancer app to each study participant. We can, therefore, not adjust for this in the analyses and come closer to whether changes in QoL are associated with the adolescents and young adults cancer app or other external factors. Especially in the treatment group, external factors such as medical oncological treatments with accompanying side effects, supportive care medication, and other health care interventions might interfere with QoL.

Further, if we had included a control group in the study, the impact of the app on QoL would have been better elucidated.

A bias might be that patients for the study were recruited and helped to download the adolescents and young adults cancer app by youth coordinators. The contact with these health care professionals might also have influenced QoL. Furthermore, results might have been different if the use was prompted by health care professionals throughout the cancer trajectory and integrated as part of the clinical care.

Originally, it was planned to include 100 patients [14]. Due to the global pandemic with COVID-19, recruitment was ended at 85 participants. However, the estimated geographical distribution was maintained.

Other limitations might be selection bias and missing data, which could influence the internal validity and generalizability of our findings.

A strength of this study is the nationwide design, including adolescents and young adults with cancer from all regions in Denmark with a broad variation in diagnosis and age distribution. The study is also strengthened because the adolescents and young adults cancer app was thoroughly developed in cocreation with adolescent and young adult patients aiming to improve QoL [5,14,15].

### Further Implications

This study and previous findings [40] on the impact of this adolescents and young adults cancer app on adolescent and young adult patients’ lives have led to the decision to implement the adolescents and young adults cancer app on a national level in Demark. However, the effect on self-empowerment and self-efficacy of using the app, as well as the impact on different functionalities of the app on QoL, remain to be investigated further. Furthermore, it must be acknowledged that apps are dynamic tools that need continuous development and adaptation to new challenges.

### Conclusions

In this study, we found an improvement in specific QoL scales for both adolescents and young adults in treatment and adolescents and young adults in follow-up when using the app for 6 weeks.

The global health or overall QoL score improved significantly in the follow-up group. In the treatment group, it remained stable.

## Abbreviations

EORTC QLQ-C30: European Organization for Research and Treatment of Cancer Quality of Life Questionnaire Core 30
PRO: patient-reported outcome
QoL: quality of life

## Data Availability

The data sets generated and analyzed during this study are not publicly available due to the Danish data protection rules but could be available from the corresponding author on reasonable request.
